# The Comparison of Plasma and Cerebrospinal Fluid R(−)- and S(+)-Flurbiprofen Concentration After Intravenous Injection of Flurbiprofen Axetil in Human Subjects

**DOI:** 10.3389/fphar.2021.646196

**Published:** 2021-04-30

**Authors:** Han Yao, Xingxian Luo, Hong Zhang, Haiyan An, Wanyu Feng, Yi Feng

**Affiliations:** ^1^ Department of Anesthesiology, Peking University People’s Hospital, Beijing, China; ^2^ Department of Pharmacy, Peking University People’s Hospital, Beijing, China

**Keywords:** flurbiprofen axetil, R(-)-flurbiprofen, S(+)-flurbiprofen, cerebrospinal fluid, plasma

## Abstract

**Background:** Flurbiprofen axetil is a prodrug that releases the active substance through enzymatic removal of the ester moiety. It is formulated through encapsulation in a lipid microsphere carrier, and widely used to treat perioperative pain. Here, we studied the distribution of R (−)- and S (+)-flurbiprofen in human plasma and cerebrospinal fluid (CSF) after intravenous injection of flurbiprofen axetil.

**Methods:** A total of 70 adult patients undergoing elective lower limb surgery under spinal anesthesia were given a single intravenous injection of 100-mg flurbiprofen axetil. The patients were randomly assigned to 10 groups for plasma and CSF sampling at 10 time points (5–50 min) after subarachnoid puncture and before actual spinal anesthesia. R (−)- and S (+)-flurbiprofen and CSF/plasma ratio were determined by liquid chromatography–tandem mass spectrometry.

**Results:** R (−)-flurbiprofen concentration ranged from 2.01 to 10.9 μg/mL in plasma and 1.46–34.4 ng/mL in CSF. S (+)-flurbiprofen concentration ranged from 1.18 to 10.8 μg/mL in plasma and from 2.53 to 47 ng/mL in CSF. In comparison to S (+)-flurbiprofen, R (−)-flurbiprofen concentration was significantly higher in plasma at all time points (*p* < 0.05) except at 30 or 40 min, and lower in CSF at all time points (*p* < 0.05) except at 10, 15 and 40 min. Analysis after correcting drug concentration for body mass index also revealed higher plasma and lower CSF R (−)-flurbiprofen concentration. In comparison to S (+)-flurbiprofen, AUC_0–50_ for R (−)-flurbiprofen was larger in plasma and smaller in CSF (*p* < 0.05 for both), and accordingly smaller CSF/plasma AUC_0–50_ ratio (*p* < 0.05). There was a positive correlation between R (−)-flurbiprofen concentration and S (+)-flurbiprofen concentration in plasma (r = 0.725, *p* < 0.001) as well as in CSF (r = 0.718, *p* < 0.001), and a negative correlation between plasma and CSF concentration of S (+)-flurbiprofen (r = −0.250, *p* = 0.037), but not R (−)-flurbiprofen.

**Conclusion:** Distribution of R (−)- and S (+)-flurbiprofen in plasma and CSF differed significantly. Penetration of R (−)-flurbiprofen into the CNS was lower than S (+)-flurbiprofen.

## Introduction

Non-steroidal anti-inflammatory drugs (NSAIDs) are major component of perioperative multimodal analgesia and play an important role in the implementation of enhanced recovery after surgery. NSAIDs inhibit cyclooxygenase (COX) peripherally. However, a number of studies suggested that some effects of NSAIDs may be mediated by their action in the central nervous system (CNS) (Burian and Geisslinger, 2005; Ortiz et al., 2008). Clinical studies have found increased prostaglandin E_2_ (PGE_2_) concentrations in cerebrospinal fluid (CSF) in patients undergoing total hip replacement surgery, which positively correlate with postoperative pain, suggesting that the inflammation-induced up-regulation of PGE_2_ in CSF may affect clinical prognosis (Buvanendran et al., 2006). Peripheral inflammation can up-regulate COX expression at a protein level in the CNS (Samad et al., 2001), leading to increased PGE_2_ levels in CSF and ultimately to peripheral hyperalgesia. Intrathecal injection of COX-2 inhibitors into animals can inhibit COX-2 in CNS, reversing the up-regulation of PGE_2_ in CSF and thereby preventing pain (Mehta et al., 2008). However, ketoprofen, a non-selective COX inhibitor, showed <0.6% penetration into the CSF (Mannila et al., 2006). The low penetration of NSAIDs appears to depend on their lipophilicity (Matoga et al., 1999).

Flurbiprofen axetil, 1-acetoxyethyl 2-(2-fluoro-[1,1′-biphenyl]-4-yl)propanoate, is a non-selective COX inhibitor commonly used in clinical practice as an antipyretic and analgesic drug. Unlike the active form of flurbiprofen, which is poorly soluble in water (5–10 μg/mL), flurbiprofen axetil is highly lipophilic and can be encapsulated into lipid microspheres (between the outer layer of lecithin and the inner fatty oil matrix). Flurbiprofen has been shown to penetrate the blood-brain barrier, and could be detected at 5 min to 18 h after systemic administration (Kumpulainen et al., 2010; Zhang et al., 2017). This raises the possibility that the drug exerts its effects, at least partly, by influencing the CNS. Specifically, esterification of flurbiprofen to flurbiprofen axetil increases its lipophilicity and improves penetration through the blood–brain barrier.

A related question is which of the enantiomeric forms of flurbiprofen, S (+) or R (−), may be present at higher concentrations in the CNS and circulation, as the two enantiomers differ in their pharmacodynamic characteristics (Geisslinger et al., 1994a; Malmberg and Yaksh, 1994; Geisslinger et al., 2000). However, to the best of our knowledge, the distribution of the two flurbiprofen isomers in CSF and plasma in patients receiving flurbiprofen axetil has not yet been examined.

Flurbiprofen axetil has two chiral carbon atoms (one in the flurbiprofen part and one in the acetoxyethyl part), and consequently two pairs of diastereoizomers and four enantiomers. Except naproxen, all commercially available NSAIDs based on 2-arylpropionic acid are racemic mixtures with approximately 1:1 ratio (Caldwell et al., 1988).

In the present study, patients who planned to undergo spinal anesthesia received a single intravenous injection of 100-mg flurbiprofen axetil. Plasma and CSF samples were collected at 10 time points at 5–50 min to determine R (−)- and S (+)-flurbiprofen using liquid chromatography-tandem mass spectrometry (LC-MS/MS).

## Methods

### Patients

This study was approved by Ethical Review Committee of Peking University People’s Hospital (#2019PHB169–01) and registered in the ClinicalTrials.gov database (NCT04128410). The study plan was explained to the patients before surgery, and written informed consent was obtained prior to enrollment.

Adult patients (18–85 years of age) scheduled for selective joint replacement under spinal anesthesia between October 2019 and June 2020 at Peking University People’s Hospital in Beijing, China (n = 105) were eligible. Patients were excluded if they had American Association of Anesthesiology physical status of three or higher, asthma, liver or kidney dysfunction, peptic ulcer, or allergy to NSAIDs; if they had received NSAIDs, CYP2C9 inhibitors (e.g., cimetidine, amiodarone, fluconazole, ketoconazole, and voriconazole) and inducers (e.g., rifampicin and barbiturates) within two weeks prior to surgery; or if they had abnormally low total plasma protein or albumin. Patients were excluded from data analysis if any of the following occurred after drug administration: 1) failure to perform subarachnoid puncture, 2) failure to collect CSF or venous blood samples on time, 3) failure to collect CSF samples; 4) contamination of CSF specimens by blood, or 5) hemolysis of plasma samples.

### Patient Treatment and Sample Collection

Before and during the procedures, patients were monitored by electrocardiography, blood pressure, and pulse oxygen saturation *via* an upper-extremity vascular access. Before spinal anesthesia, all patients received intravenous midazolam (1 mg), and then a single bolus of 100-mg flurbiprofen axetil (5050 E, Beijing Tide Pharmaceutical, Beijing, China) in 10 mL at a rate of 2 mL/min. Plasma and CSF samples were collected every 5 min for up to 50 min after the infusion of flurbiprofen axetil. Blood samples were obtained at the other upper-extremity median cubital vein. The patients were block-randomized into 10 groups of seven patients each, and each group was sampled at a different time point: the group sampled at 5 min after the end of infusion was denominated T5; the group sampled at 10 min, T10; and so on up to 50 min.

Blood and CSF were sampled prior to subarachnoid injection of local anesthetics. For the collection of CSF samples, subarachnoid puncture was performed 10 min prior to the scheduled sampling. After successful puncture, 1 mL CSF was collected into 2-mL sterile syringe. Patients then received 0.5% ropivacaine (15–20 mg) at the L2-3 or L3-4 space for spinal anesthesia. For the collection of plasma samples, venous blood (2 mL) was collected into a heparin anticoagulation tube. Plasma samples were stored at −80°C prior to assays.

### Stereospecific Assay of Flurbiprofen in Plasma and CSF

Simultaneous determination of R (−)- and S (+)-flurbiprofen in plasma and CSF was achieved using liquid chromatography-tandem mass spectrometry (LC-MS/MS) based on an electrospray ionization source in negative mode (Ye et al., 2013). Briefly, S-ketoprofen (internal standard, IS), R (−)-and S (+)-flurbiprofen were separated on a CHIRALPAK-IG3 column (250 × 4.6 mm, 5 μm) with isocratic elution using an acetonitrile/ammonium formate buffer [90:10 (v/v)] as mobile phase with 0.4 mL/min at 25°C. Quantitation was performed in multiple reaction monitoring mode with transitions of m/z 253.1→209.1 for IS and m/z 243.1→199.1 for R (−)- and S (+)-flurbiprofen (Supplementary Material).

The lower limit of quantitation (LLOQ) was 0.1 μg/mL and 1 ng/mL in plasma and CSF, respectively. The calibration standard curve was linear within the range of 0.1–10.0 μg/mL for plasma samples, and within the range of 1–100 ng/mL for CSF. The retention time was approximately 9.0, 10.8, and 9.0 min for R (−)-flurbiprofen, S (+)-flurbiprofen and IS, respectively. Representative chromatograms are shown in Supplementary Figure S2 (plasma) and Supplementary Figure S3 (CSF) (Supplementary Material). The methods developed for R (−)- and S (+)-flurbiprofen in both plasma and CSF met the standards by the United States Food and Drug Administration guidelines for accuracy, precision, recovery, linearity, stability, matrix effects, dilution integrity, and selectivity (FDA, 2018). Detailed information of LC-MS/MS assay is shown in Supplementary Figure S1; Supplementary Tables S1–5 of Supplementary Material.

### Statistical Analysis

Statistical analysis was performed using SPSS 25.0 software (IBM, Chicago, IL, United States). Continuous variables with normal distribution are reported as mean ± SD, and analyzed with Student’s t-test. Continuous variables not following normal distribution were analyzed with Wilcoxon signed rank test. Correlation between parameters was examined using Spearman rank correlation analysis. *p* < 0.05 was considered statistically significant. Pharmacokinetic modeling was conducted with Drug and Statistics (DAS, version 2.0, Mathematical Pharmacology Professional Committee of China, Shanghai, China) using a one-compartment model (Zhang et al., 2018).

## Results

**SCHEME 1:**
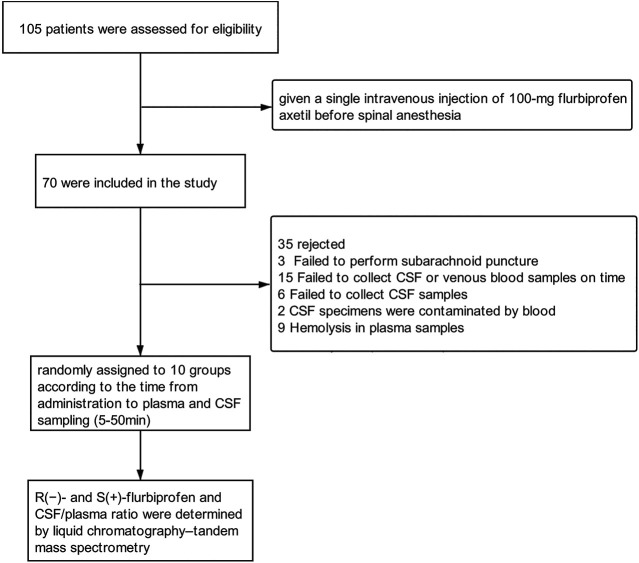
Flowchart of patient enrollment and analysis.

A total of 70 patients (70.6 ± 5.5 years of age; 11 men) were enrolled (Scheme 1, Table 1). The 10 groups were generally comparable in age, sex and BMI.

**TABLE 1:** Demographic data of all patients (n = 70) included in this study.

Variable	Group										
	T5	T10	T15	T20	T25	T30	T35	T40	T45	T50	Total
Age (years)	72.4 ± 6.8	70.1 ± 6.3	71.3 ± 4.7	70.4 ± 6.5	71.6 ± 7.2	71.1 ± 5.2	69.6 ± 4.8	71.0 ± 3.3	67.9 ± 7.3	70.9 ± 4.5	70.6 ± 5.5
BMI (kg/m^2^)	26.4 ± 6.5	26.6 ± 4.5	26.8 ± 3.3	28.1 ± 3.3	27.1 ± 1.8	26.5 ± 2.8	28.6 ± 2.8	29.2 ± 2.6	28.5 ± 4.4	26.4 ± 4.1	27.4 ± 3.7
F:M ratio	7:0	4:3	7:0	4:3	5:2	5:2	7:0	7:0	6:1	7:0	59:11

F, female; M, male; BMI, body mass index. Data presented as mean ± SD or numbers.

**FIGURE 1:**
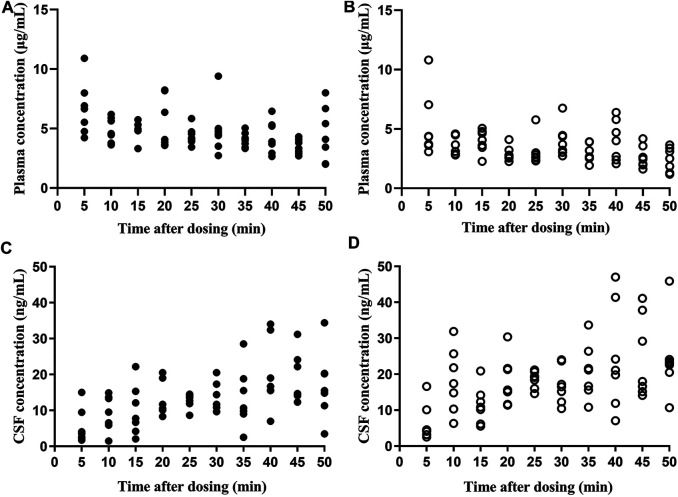
Concentrations of **(A,C)** R (−)-flurbiprofen and **(B,D)** S (+)-flurbiprofen in plasma or cerebrospinal fluid (CSF) at 5–50 min after intravenous administration of 100-mg flurbiprofen axetil. Levels were measured in seven patients at each time point.

**FIGURE 2:**
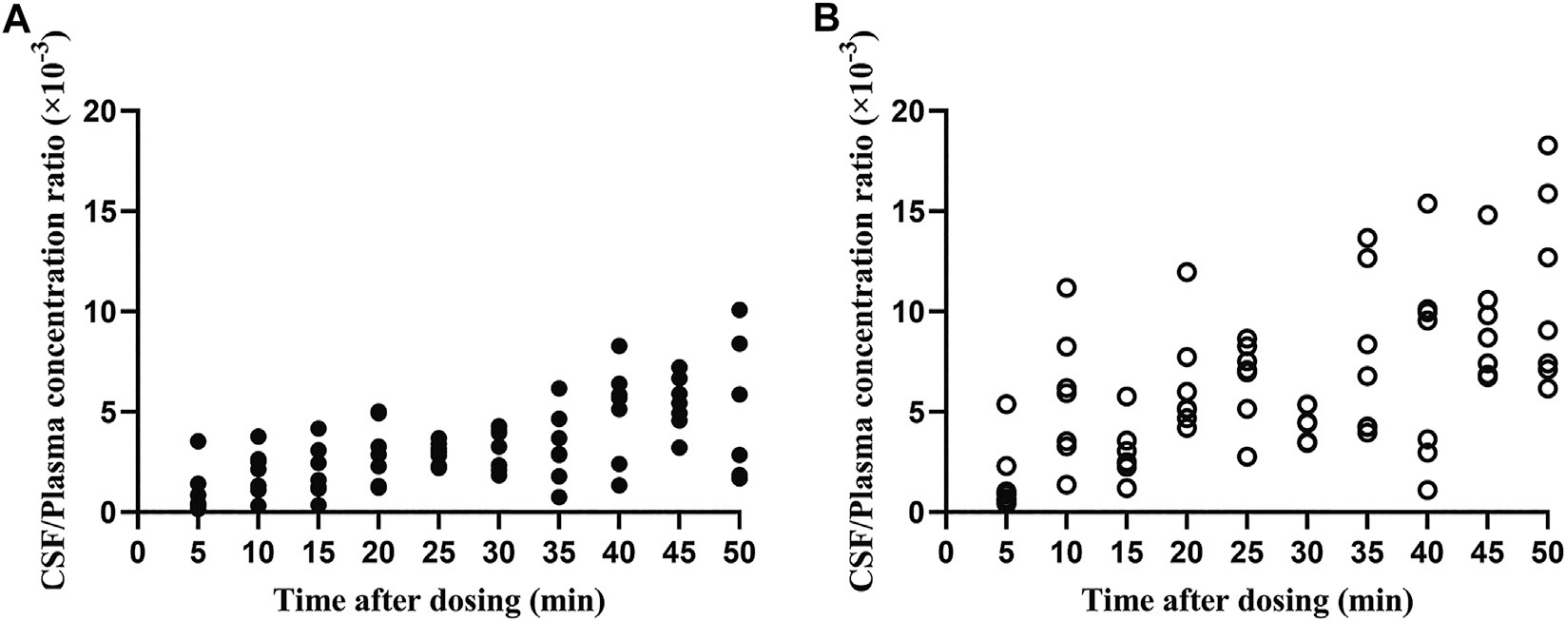
Ratios of cerebrospinal fluid (CSF)/plasma concentrations of **(A)** R (−)-flurbiprofen and **(B)** S (+)-flurbiprofen 5–50 min after intravenous administration of 100-mg flurbiprofen axetil. Levels were measured in seven patients at each time point.

Both flurbiprofen stereoisomers were detected in the plasma and CSF samples of all patients at all time points after administration. Plasma R (−)-flurbiprofen concentration was in the range of 2.01 (T50)–10.9 (T5) μg/mL (Figure 1A **)**; S (+)-flurbiprofen ranged between 1.18 (T50) and 10.8 (T5) μg/mL (Figure 1B). CSF R (−)-flurbiprofen concentration was in the range of 1.46 (T10)–34.4 (T50) ng/mL (Figure 1C); S (+)-flurbiprofen ranged between 2.53 (T5) and 47 (T40) ng/mL (Figure 1D). CSF/plasma ratio was between 0.16 × 10^–3^ (T5) and 10.10 × 10^–3^ (T50) for R (−)-flurbiprofen (Figure 2A), between 0.42 × 10^–3^ (T5) and 18.29 × 10^–3^ (T50) for S (+)-flurbiprofen (Figure 2B).

**FIGURE 3:**
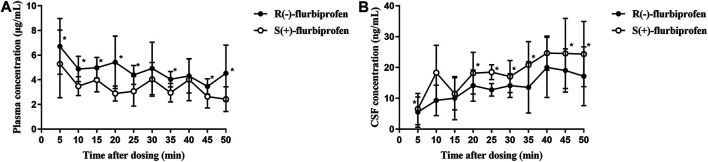
Concentrations of R (−)- and S (+)-flurbiprofen in **(A)** plasma and **(B)** cerebrospinal fluid (CSF) at 5–50 min after intravenous injection of 100-mg flurbiprofen axetil. Levels were measured in seven patients at each time point. *p < 0.05.

**FIGURE 4:**
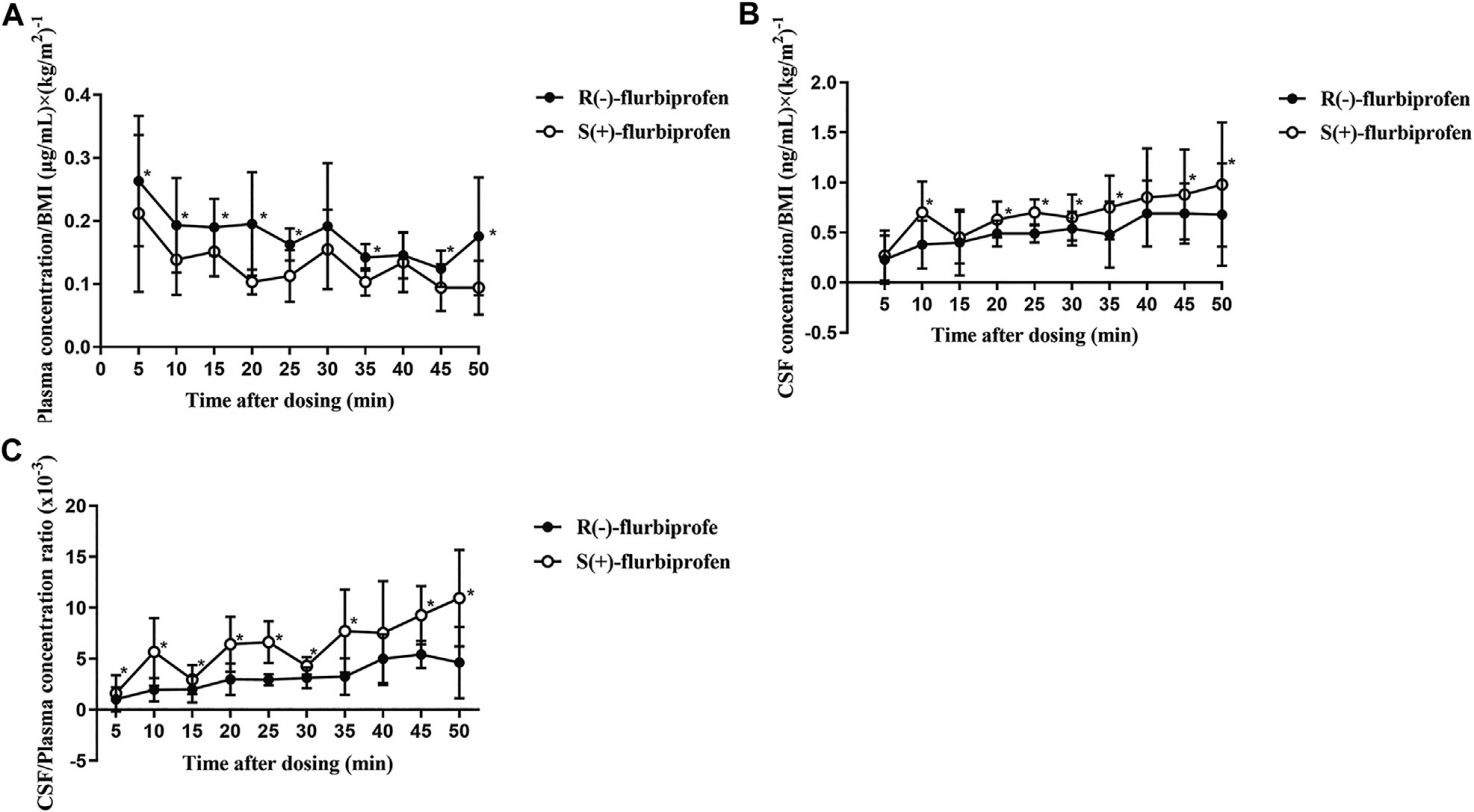
BMI-corrected concentrations of R (−)- and S (+)-flurbiprofen in **(A)** plasma and **(B)** cerebrospinal fluid (CSF) at 5–50 min after intravenous injection of 100-mg flurbiprofen axetil **(C)** Ratios of CSF/plasma concentrations of R (−)- and S (+)-flurbiprofen 5–50 min after intravenous administration of 100-mg flurbiprofen axetil. Levels were measured in seven patients at each time point. *p < 0.05.

In comparison to S (+)-flurbiprofen, R (−)-flurbiprofen concentration was higher in plasma at all time points (*p* < 0.05) except at 30 or 40 min, and lower in CSF (*p* < 0.05) except at 10, 15 and 40 min. Consistently, AUC_0–50_ for R (−)-flurbiprofen was higher in plasma [4.10 ± 0.45 vs. 3.04 ± 0.54 (μg/mL) × *h*; *p* = 0.002) and lower in CSF [10.77 ± 1.66 vs. 14.62 ± 2.05 (ng/mL) × *h*; *p* = 0.002) (Figure 3A,B). Data analysis after BMI correction also revealed higher plasma and lower CSF R (−)-flurbiprofen concentration (Figure 4A,B).

CSF/plasma ratio was significantly lower for R (−)-flurbiprofen than for S (+)-flurbiprofen at all time points (*p* < 0.05) except at 40 min. CSF/plasma AUC_0–50_ ratio was 4.94 ± 1.06 for S (+)-flurbiprofen vs. 2.65 ± 0.46 for R (−)-flurbiprofen (*p* = 0.001; Figure 4C).

There was a positive correlation between R (−)-flurbiprofen concentration and S (+)-flurbiprofen concentration in plasma (r = 0.725, *p* < 0.001) as well as in CSF (r = 0.718, *p* < 0.001), and a negative correlation between plasma and CSF concentration of S (+)-flurbiprofen (r = -0.250, *p* = 0.037), but not R (−)-flurbiprofen.

## Discussion

The current study demonstrated the presence of both flurbiprofen stereoisomers in CSF as early as 5 min after intravenous injection of 100-mg flurbiprofen axetil. The sum of the enantiomer concentrations determined at each time point were consistent with our previous study (Zhang et al., 2017), indicating the validity of the method. Significant differences were observed between R (−)- and S (+)-flurbiprofen, either with or without BMI correction. Given that there is negligible biotransformation between R (−)- and S (+)-flurbiprofen in humans (Geisslinger et al., 1994b), these results indicate differential distribution.

The two flurbiprofen enantiomers have distinct pharmacodynamic actions: S (+)-flurbiprofen inhibits COX to a much greater extent than R (−)-flurbiprofen (Evans, 1992; Malmberg and Yaksh, 1994; Geisslinger et al., 2000) and exerts much stronger antinociceptive effects (Geisslinger et al., 1994a; Sugimoto et al., 2016). In the CNS, both enantiomers appear to have antinociceptive effects (S > R) and inhibit the release of PGE_2_ in the spinal cord (Malmberg and Yaksh, 1994; Neugebauer et al., 1995). In fact, the targeting ability of R (−)-flurbiprofen toward prostaglandin release or other pathways in the CNS, such as NF-κB signaling, may be stronger than its inhibitory activity against COX (Tegeder et al., 2001). R (−)-flurbiprofen has also been shown to alleviate endocannabinoid-mediated chronic neuropathic pain and reduce glutamate release (Bishay et al., 2010). Our results showed that both S (+)-flurbiprofen and R (−)-flurbiprofen could be detected in CSF as early as 5 min after intravenous injection, supporting the contribution of central mechanism to the analgesic action. The concentrations of both enantiomers increased over time, with the S (+)-enantiomer showing faster and greater CNS penetration. In fact, the CSF/plasma concentration ratio for S (+)-enantiomer at 50 min (1.829%) was nearly twice that of R (−)-flurbiprofen (1.010%).

Effects of flurbiprofen axetil on the CNS may help to explain reduced rate of postoperative cognitive dysfunction in patients over 70 years undergoing major surgery (Wang et al., 2019) or surgery to replace joints in the lower extremities (Memtsoudis et al., 2019). R (−)-flurbiprofen may also be effective for Alzheimer’s disease (Sozio et al., 2013; Gulcicek et al., 2018) *via* inhibiting mitochondrial calcium overload caused by β-amyloid toxicity.

The maximum concentration of both flurbiprofen enantiomers in plasma occurred at 5 min and decreased thereafter, though there were small increases at 30 and 50 min, perhaps reflecting differential expression of CYP2C9 isoforms that metabolize flurbiprofen in humans (Lee et al., 2003; Zhang et al., 2007; Wang et al., 2009). In contrast, the levels of both enantiomers were initially low in CSF and increased over time, reflecting fast drug degradation by blood enzymes and relatively slow penetration of the blood–brain barrier. In the current study, we noticed opposite temporal profiles of CSF (increase) vs. plasma (decrease) concentration for both R (−)- and S (+)-flurbiprofen. A variety of mechanisms may have contributed this finding. First, enzymes that metabolizes flurbiprofen is abundant in periphery, but may be absent in CSF. A previous study showed indomethacin (also a NSAID) could bind to a high degree to proteins in CSF (Muller et al., 1991). Active transport is another possibility, but has not been found in the brain (Kumpulainen et al., 2010). Indeed, the CSF/plasma concentration ratio of the two enantiomers reached a maximum of 1–2% at 50 min after administration. Consistent with earlier entry of flurbiprofen axetil into CSF, both flurbiprofen enantiomers could be detected in CSF at 5 min post-administration. Plasma concentration of R (−)-enantiomer was higher than S-enantiomer in the current study. The inconsistency between our finding and previous studies (Jamali et al., 1988; Knadler et al., 1992) may reflect different routes of administration. In previous studies, plasma concentration of S flurbiprofen was reported to be higher using oral administration. In the current study, flurbiprofen axetil was given intravenously, and thus without the first pass effect.

The current study has several limitations. In particular, only one CSF sample could be collected from each patient for ethical reasons. Therefore, we randomly divided the patients into 10 groups according to the time of CSF sampling, and each patient underwent only one subarachnoid puncture. Although this method is not statistically ideal, we have previously used it to obtain valuable information (Zhang et al., 2017). Furthermore, we measured total, but not free R (−)- and S (+)-flurbiprofen. Blood enzymes rapidly hydrolyze flurbiprofen axetil to release flurbiprofen into plasma, but we did not measure free flurbiprofen levels. A previous study suggested that the pharmacodynamics of flurbiprofen could be influenced by serum albumin concentration (Ohmukai, 1996). Therefore, PK-PD of the free R (−)- and S (+)- flurbiprofen should be further verified. Due to clinical and ethical concerns, we did not measure drug levels beyond 50 min. Also, CYP2C9 genotyping analysis was not performed. Having said that, >90% of the Chinese subjects harbor the *1/*1 genotype, and the remaining two genotypes (*1/*3 and *1/*13) had only 4.3–7.7% and <1.2% frequency (Zhang et al., 2007). Accordingly, the bias caused by genotype imbalance is minimal. Subjects in the current study are mostly elderly, with a high female:male ratio. These characteristics are consistent with epidemiological studies (Debi et al., 2009; Elbaz et al., 2011), but may limit generalizability of our results.

In summary, the current study provided evidence for higher penetration of S (+)-flurbiprofen vs. R (−)-flurbiprofen into the CNS in human subjects.

## Data Availability

The datasets presented in this study can be found in online repositories. The names of the repository/repositories and accession number(s) can be found in the article/Supplementary Material.
