# Supplementary material for: The Comparison of Plasma and Cerebrospinal Fluid R(−)- and S(+)-Flurbiprofen Concentration After Intravenous Injection of Flurbiprofen Axetil in Human Subjects
Source: Front Pharmacol. 2021 Apr 30;12:646196. doi: 10.3389/fphar.2021.646196 (PMC8120306; doi:10.3389/fphar.2021.646196)
Supplement: Supplementary file 1 [file DataSheet1.PDF]

Supplementary Material

Simultaneous determination of R(−)- and S(+)-flurbiprofen in human plasma and cerebrospinal fluid by LC-MS/MS

1. Materials and methods

1.1 Chemicals and materials

R(−)- and S(+)-flurbiprofen (purity>98%) were purchased from Cayman Chemical (Michigan, USA). S-ketoprofen (purity>99%) was used as the internal standard (IS), offered by the National Institute for the Control of Pharmaceutical and Biological Products (Beijing, China). LC-MS grade acetonitrile was bought from Merck Millipore (Darmstadt, Germany). LC-MS grade formic acid was used from Sigma (St. Louis, MO, USA). The blank samples of plasma and cerebrospinal fluid (CSF) were collected from six healthy volunteers. The laboratory used the Milli-Q purification system from Milliporel to prepare purified water. All other experiment chemicals were with analytical grade.

1.2 Preparation of stock solutions

Methanol, as dissolvant, was used to dilute and prepare all the stock solutions (1 mg/mL). The stock solutions were diluted to the working solutions respectively containing 500 μg/mL of R(−)- and S(+)-flurbiprofen with methanol. The IS working solutions for plasma (1 μg/mL of S-ketoprofen) and CSF (100 ng/mL of S-ketoprofen) were also prepared with methanol.

1.3 Instrumentations and chromatographic conditions

Agilent 1200 HPLC with G1329A Sampler (Agilent, USA) equipped with QTRAP 5500 triple quadrupole mass spectrometer (Applied Biosystem, USA) was used to analyze these agents. R(−)- and S(+)-flurbiprofen were separated using a CHIRALPAK-IG3 column (250 × 4.6 mm, 5 μm) at 25 °C and an injection volume of 5 μL. Isocratic elution was employed for the mobile phase

composed of 90% acetonitrile and 10% ammonium formate buffer (10 mM) with a pH of 3 with formic acid. The autosampler temperature was set at 4 °C and the total run time of the analytes was 12 min with 0.4 mL/min flow rate. The mass spectrometric quantitation was performed by LC-MS/MS equipped with electrospray ionization interface in a negative mode. Quantitation was measured in multiple reaction monitoring mode with transitions of m/z 253.1→209.1 for IS and m/z 243.1→199.1 for R(−)- and S(+)-flurbiprofen. The detailed measurement parameters of R(−)-flurbiprofen, S(+)-flurbiprofen and IS were described in Table S-1. Chromatogram information collection and data processing were performed by using Analyst 1.6.1 Data processing software.

2. Standard and sample preparation

2.1 Calibration standards and quality control samples

The different working solutions for calibration and quality control (QC) were achieved with methanol by the gradient dilution of the stock solutions. The calibration standards for R(−)- and S(+)-flurbiprofen were prepared by blank plasma to the final concentration covered by seven points as follow: 0.1, 0.25, 0.5, 1, 2.5, 5, 10 μg/mL. QC samples in plasma were prepared similarly to the various concentration of 0.2, 1 and 8 μg/mL for R(−)- and S(+)-flurbiprofen. In addition, the calibration standards for R(−)- and S(+)-flurbiprofen in CSF were performed as follow: 1, 2.5, 5, 10, 25, 50, 100 ng/mL. QC samples in CSF were set 2, 10 and 80 ng/mL for R(−)- and S(+)-flurbiprofen.

2.2 Sample preparation

For the plasma samples, a 200 μL aliquot of plasma, 20 μL of IS working solution (1 μg/mL) and 600 μL of acetonitrile as the precipitated protein were added in a 1.5 mL Eppendorf tube. After the mixture samples were vortexed for 5 min and centrifuged (13400 × g, 4 °C) for 10 min, an aliquot of 5 μL of the supernatant was injected into the LC-MS/MS instrument for analysis.

For the CSF samples, a 400 μL aliquot of CSF, 40 μL of IS working solution (100 ng/mL) and 800 μL of ethyl acetate were added together to extract. After the mixture was vortexed for 5 min and

centrifuged (13400 × g, 4 °C) for 5 min, 700 μL of supernatant was extracted and blew dry with nitrogen. Finally, after 100 μL of reconstituted solution composed of 90% acetonitrile and 10% H_2O was added and centrifuged (13400 × g, 4 °C) for 10 min, and then an aliquot of 5 μL of the supernatant was injected into the LC-MS/MS instrument for analysis.

3. Method validation

3.1 Selectivity

The endogenous interference was evaluated by blank plasma and CSF samples of six individual human at the retention times of the R(−)-flurbiprofen, S(+)-flurbiprofen and IS in the chromatograms. Each blank sample was tested for interference and compared to the lower limit of quantification (LLOQ) of R(−)- and S(+)-flurbiprofen.

3.2 Accuracy and precision

Four levels of QC samples (0.1, 0.2, 1 and 8 μg/mL for plasma; 1, 2, 10 and 80 ng/mL for CSF) were consecutively using six replicates assayed during a single analytical run to assess the intra-day accuracy and precision of R(−)- and S(+)-flurbiprofen, while the inter-day accuracy were performed by calculation on three consecutive validation days. Accuracy was expressed by RE% (percentage of the relative error), whereas precision was expressed by RSD% (percentage of the relative standard deviation). The acceptability criterion for both accuracy and precision within ± 15% for RE% and RSD% were required except LLOQ within ± 20%.

3.3 Linearity and sensitivity

The calibration curves were constructed by plotting the peak area ratios (y) of R(−)- and S(+)-flurbiprofen to IS versus theoretical concentrations (x), through weighted least-squares linear regression analysis with a weighting factor of $1/x^2$. The LLOQ of R(−)- and S(+)-flurbiprofen for plasma and CSF as the lowest concentration of the calibration curves was employed to assess the sensitivity, which required signal-to-noise ratio (S/N) >10.

3.4 The extraction recovery and matrix effect

R(−)- and S(+)-flurbiprofen were conducted using QC samples at three levels. The extraction recovery was defined as the chromatographic peak area of pre-extracted QC samples compared to those obtained with post-extracted QC samples in six replicates. The matrix effect was evaluated by comparing the peak area of the standard spiking in the extracted blank sample concentration with those of neat solutions at the respective concentration levels.

3.5 Dilution integrity

Dilution of highly concentrated plasma samples, with concentrations beyond the linearity range of the proposed method, was evaluated for its effect on determination of R(−)- and S(+)-flurbiprofen plasma concentration. Plasma samples spiked with high concentrations of R(−)- and S(+)-flurbiprofen (20 μg/mL) were used dilution folds (1:4) with blank plasma samples. Diluted samples were then treated as under "Sample preparation". Following actual analysis, the found concentrations were calculated for each sample and then compared to the nominal values to calculate RE%.

3.6 Stability tests

R(−)- and S(+)-flurbiprofen in plasma and CSF were studied under four different conditions, including 3 freeze-thaw cycles (from -80 °C to ambient temprature), the processed sample in autosampler (at -4 °C), short-term (at ambient temperature for 6 h) and long term stability (stored at -80 °C for 14 days) were investigated with six replicates. The stability was regarded as acceptability if the average percentage concentration were within 85-115% of the initial concentration.

4. Results

4.1 Selectivity

Product ion mass spectra of R(−)-flurbiprofen (243.1→199.1), S(+)-flurbiprofen (243.1→199.1) and IS (253.1→209.1) with negative ionization mode were shown in Figure S-1. No endogenous

substances interfered with R(−)-flurbiprofen, S(+)-flurbiprofen and IS was observed both for plasma and CSF samples. The retention times of R(−)-flurbiprofen, S(+)-flurbiprofen and IS approximately were 9.0, 10.8, and 9.0 min, respectively. For the typical chromatograms of plasma, the blank plasma sample, the blank plasma sample spiked R(−)-flurbiprofen (1 μg/mL), S(+)-flurbiprofen (1 μg/mL) and IS (1 μg/mL), and a plasma sample of 15 min after injected intravenously with a single injection of 100-mg flurbiprofen axetil were shown in Figure S-2. For the typical chromatograms of CSF, the blank CSF sample, the blank CSF sample spiked R(−)-flurbiprofen (10 ng/mL), S(+)-flurbiprofen (10 ng/mL) and IS (100 ng/mL), and a CSF sample of 15 min after injected intravenously with a single injection of 100-mg flurbiprofen axetil were shown in Figure S-3.

4.2 Accuracy and precision

As shown in Table S-2 and Table S-3, the intra-day and inter-day accuracy (RE%) of plasma and CSF QC samples at four levels ranged 3.15 to 10.22% while precision (RSD%) were between 2.13% and 11.24%.

4.3 Linearity and sensitivity

The established method used least-squares linear regression and the calibration standard curves were calculated as follows: y=0.0091x-0.0204 [r=0.997, R(−)-flurbiprofen in plasma] , y=0.0012x-0.147 [r=0.999, S(+)-flurbiprofen in plasma], y=0.0296x-0.0388 [r=0.997, R(−)-flurbiprofen in CSF] and y=0.0215x-0.0278 [r=0.997, S(+)-flurbiprofen in CSF]. The results suggested that the concentration of R(−)- and S(+)-flurbiprofen presented a good linear relationship with 0.1-10.0 μg/mL for plasma and 1-100 ng/mL for CSF. The LLOQ of R(−)- and S(+)-flurbiprofen was determined to be 0.1 μg/mL and 1 ng/mL for plasma and CSF, respectively, which was found to be sufficient for pharmacokinetic analysis of R(−)- and S(+)-flurbiprofen. The accuracy and precision of LLOQ were within 15%, indicating a fairly good sensitivity in the detection of R(−)- and S(+)-flurbiprofen in plasma and CSF.

4.4 The extraction recovery and matrix effect

Recovery and matrix effects were studied by three different levels of QC samples for plasma and CSF. As shown in Table S-4 and Table S-5, the results of the average extraction recoveries for R(−)- and S(+)-flurbiprofen were 2.32-8.32% for plasma, and 3.12-8.56% for CSF. In addition, the mean matrix effect of R(−)- and S(+)-flurbiprofen were 2.16-9.23% for plasma and 1.38-9.12% for the CSF. These results indicated that no apparent ionization interference was found to influence the determination of R(−)- and S(+)-flurbiprofen in plasma and CSF. The results were also shown in Table S-4 and Table S-5, which were fulfilled with the US FDA guidelines for the method validation.

4.5 Dilution integrity

Fold dilutions (1:4) of concentrated samples yielded acceptable recoveries with error values not more than 5% RE, suggesting that the method can be used determination of samples with concentration higher than the upper limit of the standard curve.

4.6 Stability

Stability test in plasma and CSF including bench-top freeze–thaw, auto-sampler, short-term and long-term stability studies indicated that R(−)- and S(+)-flurbiprofen was stable under various storage conditions, which were consistent with the previous study (Ye et al., 2013).

Reference

Ye, J., Yang, W., Yu, W., Sun, Y., Chen, S., Shen, Z., et al. (2013). Establishment of stereospecific assay of flurbiprofen by high-performance liquid-chromatographic tandem-mass spectrometry and its application to pharmacokinetic study. *Chinese Pharmaceutical Journal* 48**,** 1099-1103. doi: 10.11669/cpj.2013.13.014.

Table S-1. Summary of the MRM transitions for R(−)-flurbiprofen, S(+)-flurbiprofen and IS used in the LC–MS/MS analysis.

Compound	Transitions (Da)	Dwell time (ms)	DP (V)	EP(V)	CE(V)	CXP (V)
R(−)-flurbiprofen	243.1→199.1	200	-50	-10	-23	-10
S(+)-flurbiprofen	243.1→199.1	200	-50	-10	-23	-10
S- ketoprofen (IS)	253.1→209.1	200	-43	-8	-11	-15

Note: DP: declustering potential; EP: entrance potential; CE: collision energy; CXP: collision cell exit potential.

Table S-2. Precision and accuracy of R(−)- and S(+)-flurbiprofen in plasma (n=6).

Matrix	Analytes	Concentration added (μg/mL)	Intra-day		Inter-day	
			Accuracy (RE%)	Precision (RSD %)	Accuracy (RE%)	Precision (RSD %)
Plasma	R(−)-flurbiprofen	0.1	6.12	4.32	7.21	9.12
		0.2	4.23	4.78	9.34	2.13
		1	6.83	9.23	10.23	5.41
		8	9.12	6.51	6.23	8.32
	S(+)-flurbiprofen	0.1	7.23	5.21	9.01	3.21
		0.2	4.25	4.35	4.21	5.23
		1	8.2	5.82	8.12	7.21
		8	8.31	4.52	7.13	5.32

Table S-3. Precision and accuracy of R(−)- and S(+)-flurbiprofen in CSF (n=6).

Matrix	Analytes	Concentration added (ng/mL)	Intra-day		Inter-day	
			Accuracy (RE%)	Precision (RSD %)	Accuracy (RE%)	Precision (RSD %)
CSF	R(−)-flurbiprofen	1	9.13	3.52	5.23	11.24
		2	8.34	7.94	3.15	8.21
		10	5.63	3.12	9.32	4.56
		80	10.22	7.23	6.34	5.34
	S(+)-flurbiprofen	1	5.45	9.21	7.23	8.34
		2	9.56	4.12	3.18	7.56
		10	5.31	9.26	11.21	6.21
		80	7.34	8.12	9.23	4.52

Table S-4. Recovery and matrix effect of R(−)- and S(+)-flurbiprofen in plasma (n=6).

Matrix	Analytes	Concentration added (μg/mL)	Recovery (%)		Matrix effect (%)	
			Mean±SD	RSD (%)	Mean±SD	RSD (%)
Plasma	R(−)-flurbiprofen	0.2	5.23±4.12	4.78	6.21±4.12	3.13
		1	2.32±4.21	9.23	4.41±5..26	5.41
		8	8.32±6.11	6.51	9.12±6.12	8.32
	S(+)-flurbiprofen	0.2	4.71±7.32	4.21	5.12±6.38	5.23
		1	3.12±3.21	5.82	9.23±6.40	7.21
		8	4.23±8.32	4.52	2.16±5.21	5.32

Table S-5. Recovery and matrix effect of R(−)- and S(+)-flurbiprofen in CSF (n=6).

Matrix	Analytes	Concentration added (ng/mL)	Recovery (%)		Matrix effect (%)	
			Mean±SD	RSD (%)	Mean±SD	RSD (%)
CSF	R(−)-flurbiprofen	2	8.56±2.34	7.94	6.23±2.13	8.21
		10	3.12±3.24	3.12	1.38±5.12	4.56
		80	6.23±5.61	7.23	9.12±3.56	5.21
	S(+)-flurbiprofen	2	7.27±3.24	4.12	8.22±3.31	7.56
		10	5.13±5.32	11.32	6.32±6.12	6.21
		80	4.13±4.21	8.12	5.21±3.47	9.12

Figure

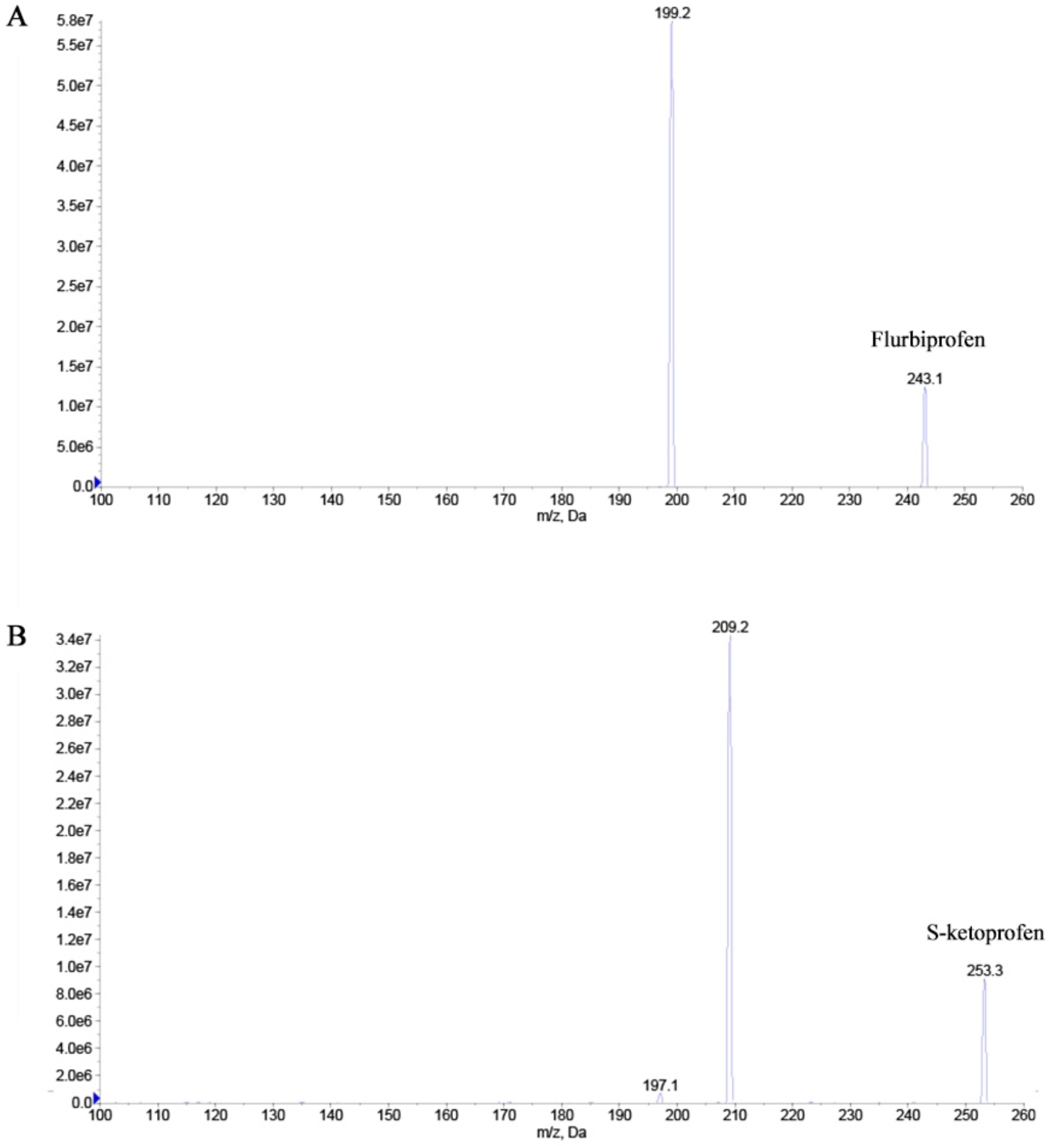

Figure S-1. Mass spectrum of flurbiprofen **(A)** and IS **(B)**.

A

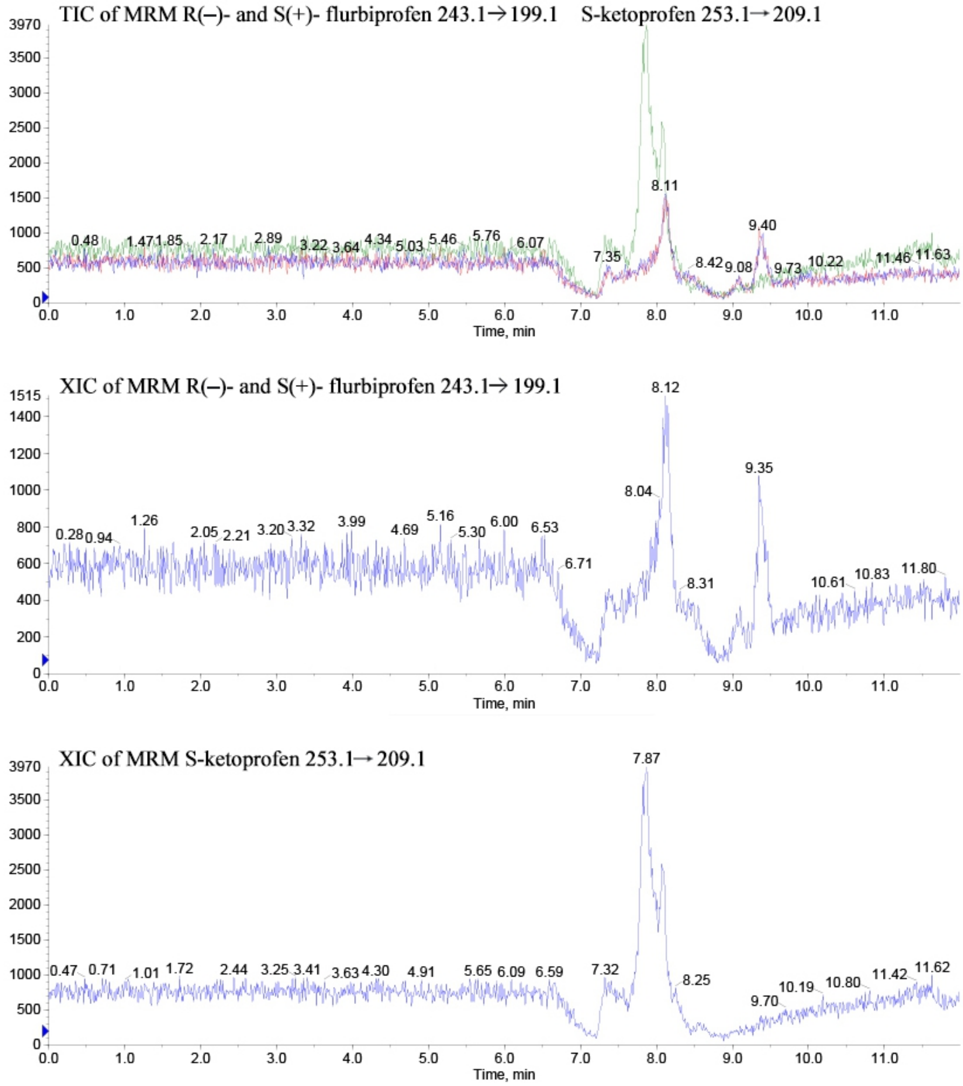
TIC of MRM R(–)- and S(+)- flurbiprofen 243.1→199.1 S-ketoprofen 253.1→209.1
8.11
9.40
7.35
Time, min
XIC of MRM R(–)- and S(+)- flurbiprofen 243.1→ 199.1
8.12
8.04
9.35
8.31
6.71
Time, min
XIC of MRM S-ketoprofen 253.1→ 209.1
7.87
7.32
8.25
9.70
Time, min

B

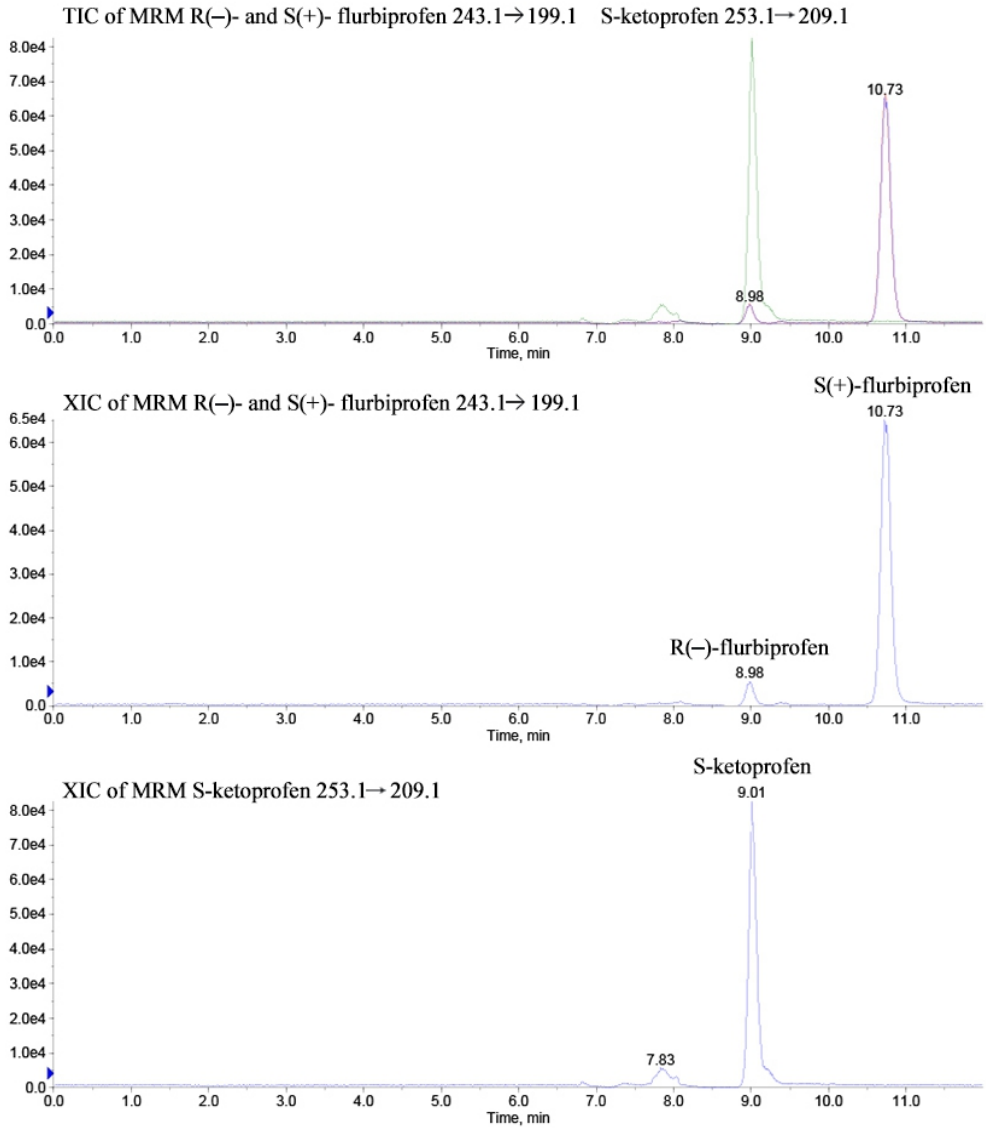
TIC of MRM R(–)- and S(+)- flurbiprofen 243.1→199.1 S-ketoprofen 253.1→209.1
10.73
8.98
Time, min
XIC of MRM R(–)- and S(+)- flurbiprofen 243.1→199.1
S(+)-flurbiprofen
10.73
R(–)-flurbiprofen
8.98
Time, min
XIC of MRM S-ketoprofen 253.1→209.1
S-ketoprofen
9.01
7.83
Time, min

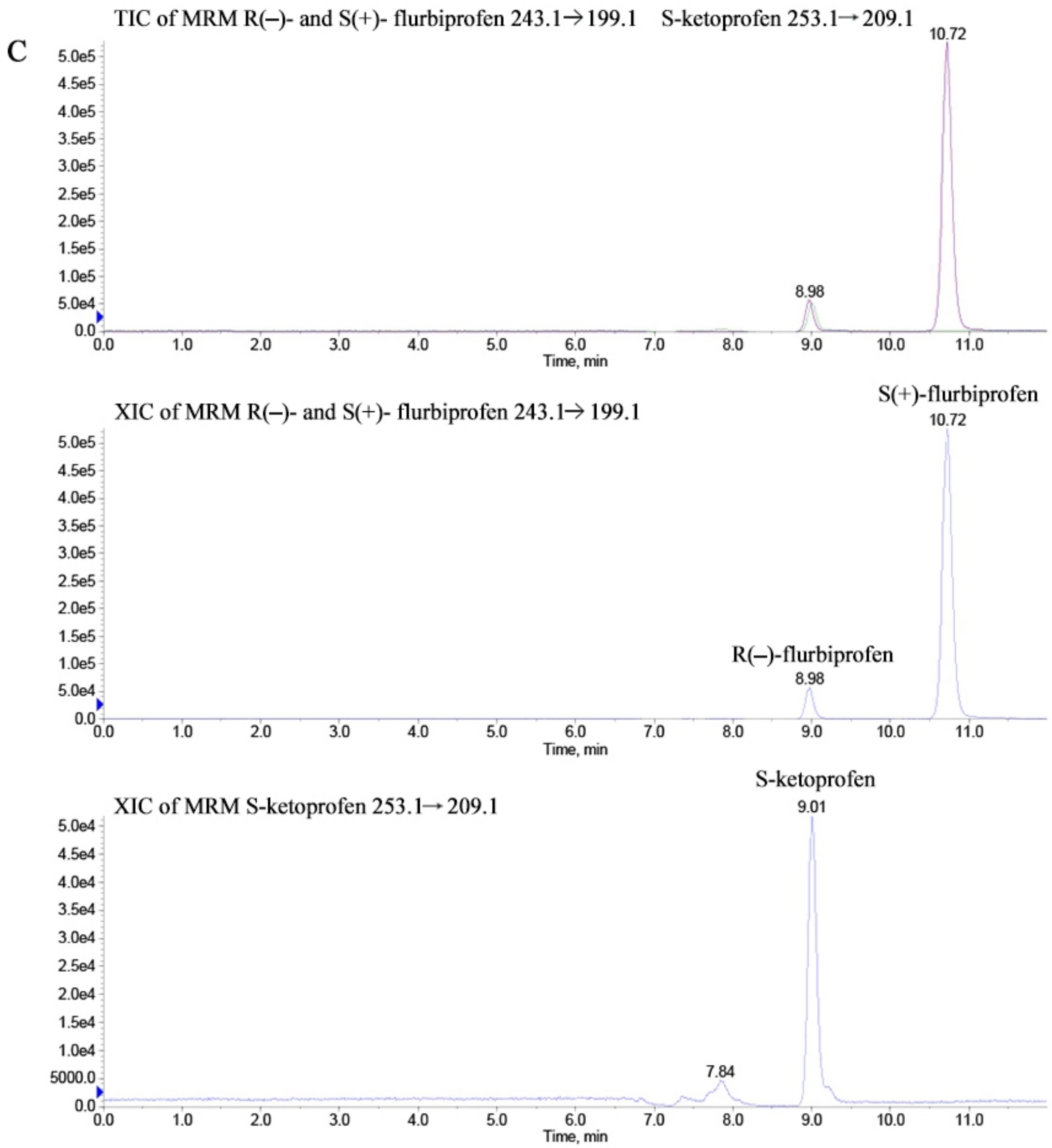

Figure S-2. Representative chromatograms of R(−)-flurbiprofen, S(+)-flurbiprofen and IS in plasma samples. **(A)** a blank plasma sample; **(B)** a blank plasma sample spiked with R(−)-flurbiprofen (1 μg/mL), S(+)-flurbiprofen (1 μg/mL) and IS (1 μg/mL); **(C)** a plasma sample of 15 min after injected intravenously with a single injection of 100-mg flurbiprofen axetil.

A

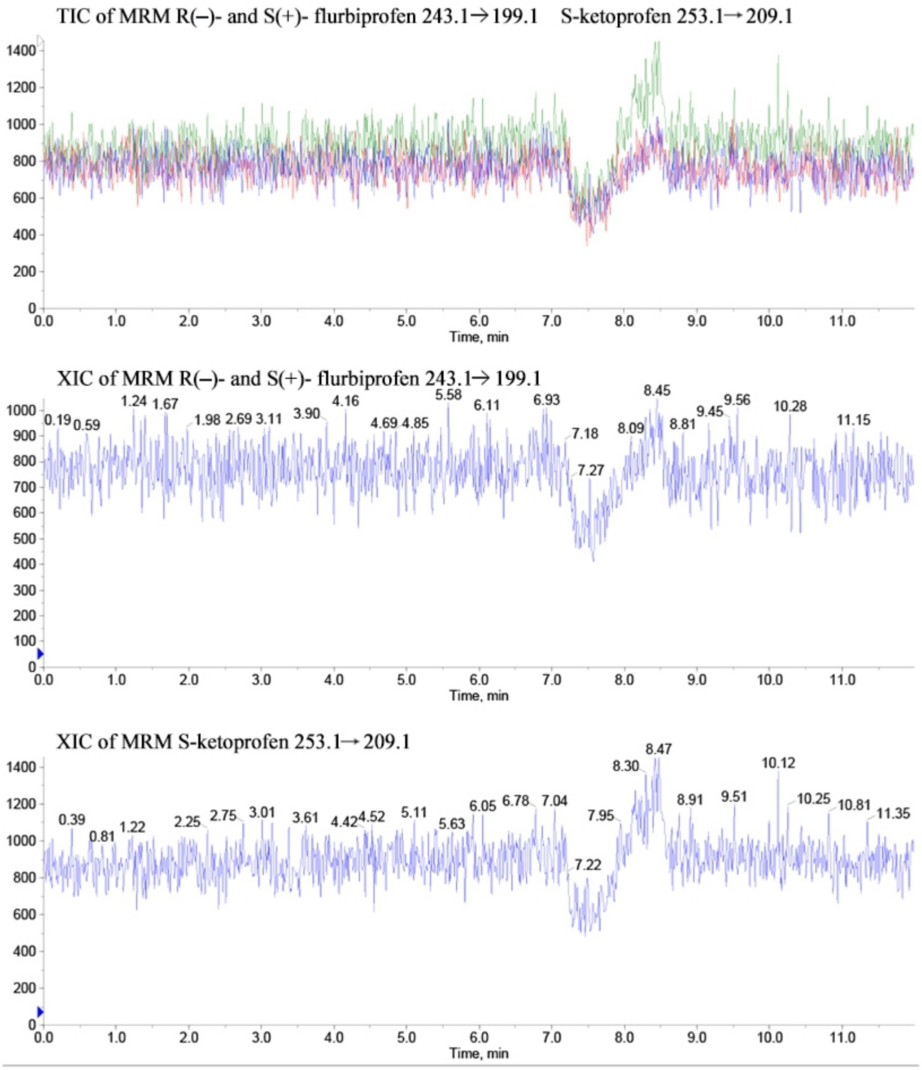
TIC of MRM R(–)- and S(+)- flurbiprofen 243.1→199.1 S-ketoprofen 253.1→209.1
Time, min
XIC of MRM R(–)- and S(+)- flurbiprofen 243.1→199.1
Time, min
XIC of MRM S-ketoprofen 253.1→209.1
Time, min

B

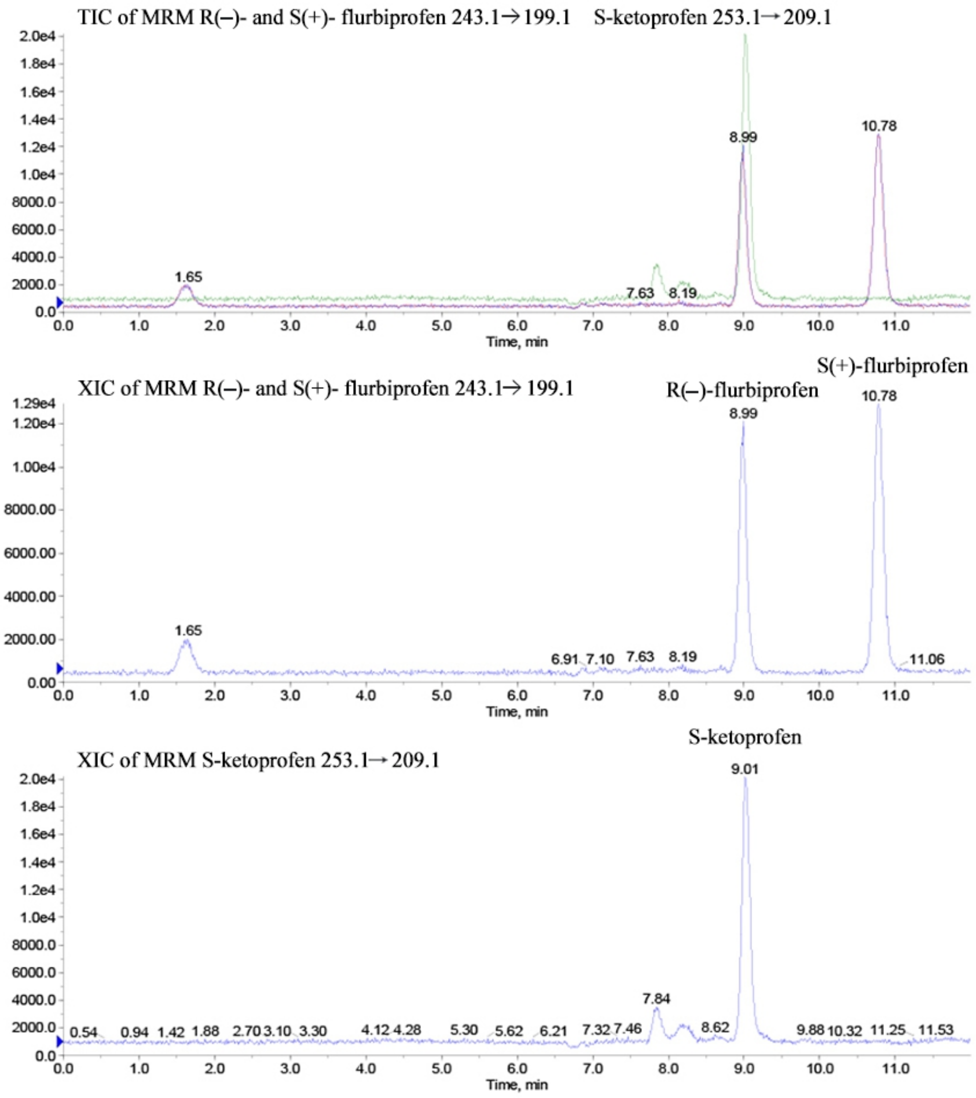
TIC of MRM R(–)- and S(+)- flurbiprofen 243.1→199.1 S-ketoprofen 253.1→209.1
8.99
10.78
1.65
7.63
8.19
Time, min
XIC of MRM R(–)- and S(+)- flurbiprofen 243.1→199.1
R(–)-flurbiprofen
S(+)-flurbiprofen
8.99
10.78
1.65
6.91 7.10 7.63 8.19
11.06
Time, min
XIC of MRM S-ketoprofen 253.1→209.1
S-ketoprofen
9.01
7.84
0.54 0.94 1.42 1.88 2.70 3.10 3.30 4.12 4.28 5.30 5.62 6.21 7.32 7.46 8.62 9.88 10.32 11.25 11.53
Time, min

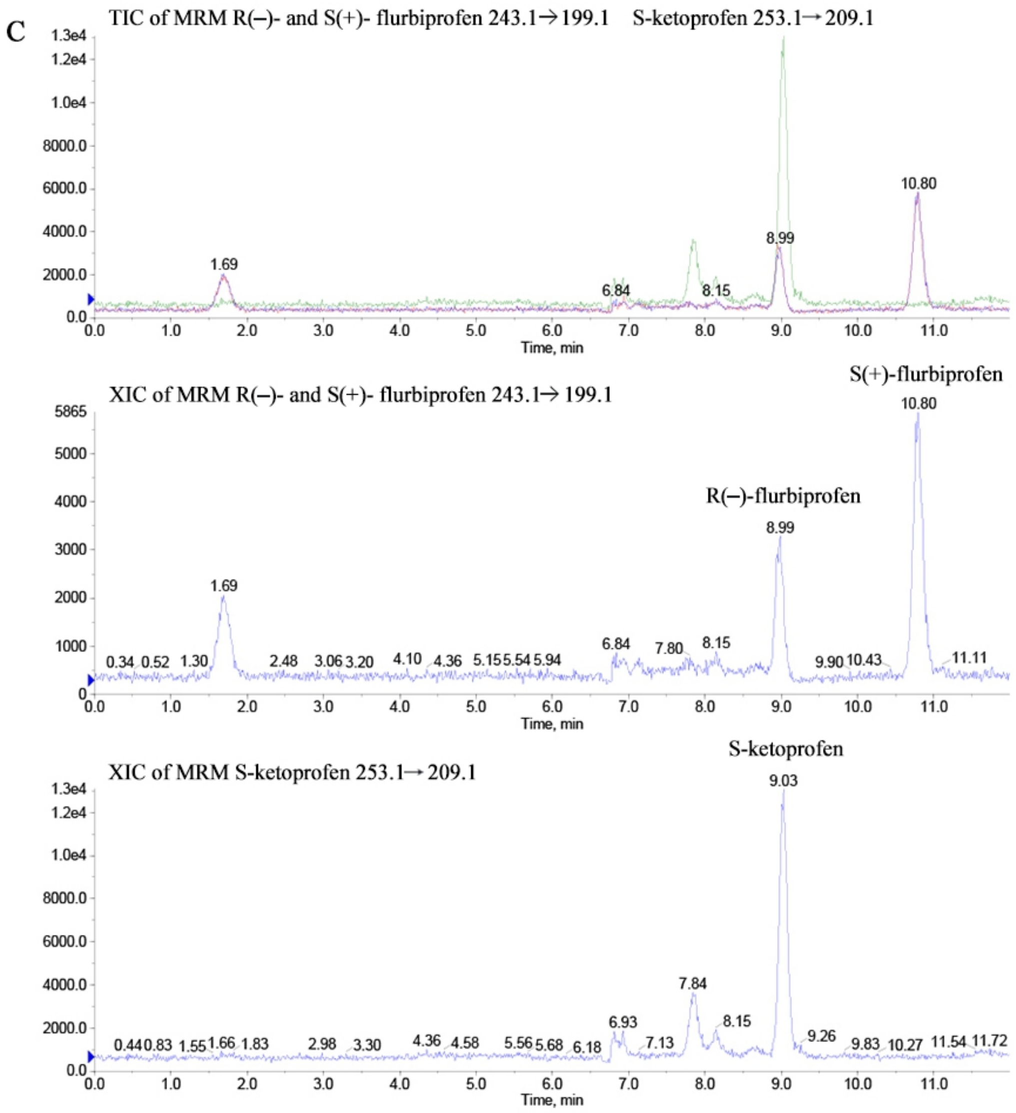

Figure S-3. Representative chromatograms of R(−)-flurbiprofen, S(+)-flurbiprofen and IS in CSF samples. **(A)** a blank CSF sample; **(B)** a blank CSF sample spiked with R(−)-flurbiprofen (10 ng/mL), S(+)-flurbiprofen (10 ng/mL) and IS (100 ng/mL); **(C)** a CSF sample of 15 min after injected intravenously with a single injection of 100-mg flurbiprofen axetil.
